# Fluid handling and blood flow patterns in neonatal respiratory distress syndrome versus transient tachypnea: a pilot study

**DOI:** 10.1186/s12887-021-03025-z

**Published:** 2021-12-03

**Authors:** Rana Ismail, Prashanth Murthy, Ayman Abou Mehrem, Zhiying Liang, Amelie Stritzke

**Affiliations:** 1 Section of Neonatology, Department of Pediatrics, Alberta Health Services, University of Calgary, Cumming School of Medicine, Calgary, Canada; 2 University of Calgary, Cumming School of Medicine, Libin Cardiovascular Institute of Alberta, Calgary, Canada

**Keywords:** Dopplers, Transient tachypnea of the newborn, Respiratory distress syndrome, Renal transition

## Abstract

**Background:**

Cardiovascular and renal adaptation in neonates with Respiratory Distress Syndrome (RDS) and Transient Tachypnea of the Newborn (TTN) may be different.

**Methods:**

Neonates ≥32 weeks were diagnosed with RDS or TTN based on clinical, radiologic and lung sonographic criteria. Weight loss, feeding, urine output, and sodium levels were recorded for the first 3 days, and serial ultrasounds assessed central and organ Doppler blood flow. A linear mixed model was used to compare the two groups.

**Results:**

Twenty-one neonates were included, 11 with TTN and 10 with RDS. Those with RDS showed less weight loss (− 2.8 +/− 2.7% versus − 5.6 +/− 3.4%), and less enteral feeds (79.2 vs 116 ml/kg/day) than those with TTN, despite similar fluid prescription. We found no difference in urine output, or serum sodium levels. Doppler parameters for any renal or central parameters were similar. However, Anterior Cerebral Artery maximum velocity was lower (*p* = 0.03), Superior Mesenteric Artery Resistance Index was higher in RDS, compared to TTN (*p* = 0.02).

**Conclusion:**

In cohort of moderately preterm to term neonates, those with RDS retained more fluid and were fed less on day 3 than those with TTN. While there were no renal or central blood flow differences, there were some cerebral and mesenteric perfusion differences which may account for different pathophysiology and management.

## Background

Neonatal respiratory distress is one of the most common clinical conditions dealt with in the Neonatal Intensive Care Unit (NICU). Within the moderate to late preterm group (32 0/7–36 + 6/7 weeks of gestation), Respiratory Distress Syndrome (RDS) was diagnosed in 7.2% of NICU admissions within the Canadian Neonatal Network (CNN) in 2016 [1]. While RDS incidence decreases with increasing gestational age (GA) [2], Transient Tachypnea of the Newborn (TTN) emerges as the most common underlying cause for respiratory distress in the term and near-term population with an estimated incidence of 3.6–5.7 per 1000 term infants [3].

While patho-physiologically, RDS and TTN represent two distinct entities, clinical overlap, especially early in the neonatal course, makes diagnosis challenging. RDS is characterized by surfactant deficiency with worsening pulmonary insufficiency over 2–3 days. TTN, in contrast, is believed to result from incomplete resorption of fluid from the newborn lung with immediate-onset tachypnea and mild work of breathing confined generally to the first 24 h of life. Radiographic imaging is employed to differentiate with a typical diffuse reticulo-granular honey-comb pattern for surfactant deficiency [2]. Lung ultrasound is a newer tool with high sensitivity and specificity to diagnose RDS early [4]. Treatment in both cases consists of respiratory support, with intubation and administration of exogenous surfactant with RDS, should respiratory support requirement exceed a certain threshold [5, 6].

In infants without RDS, independent of GA, postnatal diuresis and physiological weight loss of about 7% occur during the transitional period in the first 3 days of life [7]. Oliguria during the early stages of RDS, but not TTN, is well recognized, and urinary output peaks at around 24–48 h, just prior to improvement in pulmonary function [8, 9]. Inulin and PAH (Para-aminohippurate) clearance were markedly depressed in RDS compared to controls [10]. Onset of diuresis in infants with RDS occurred at a mean age of 27.7 h with the maximum urine output at a mean age of 44.9 h [11]. Our understanding of pathophysiologic differences between RDS and TTN, especially in terms of renal-pulmonary interaction, is limited. During the critical transitional period, volume contraction and ductal closure are the main physiologic occurrences [12]. Deepened understanding of potential differences may improve timely differentiation, clinical management, and ultimately outcomes, such as less delay in surfactant administration.

### Objectives

**T**he aim of our study is to compare fluid handling in the first 3 days in a group of moderately preterm to term infants with RDS versus TTN by comparing weight loss as primary outcome. Enteral feeding, fluid prescription, serum sodium and urinary output were compared. We assessed ultrasound-derived central and organ Doppler parameters of blood flow between the 2 groups to delineate any potential physiologic difference.

## Methods

### Study type

Single-center prospective exploratory observational pilot cohort study

### Primary outcome

Weight loss as a percentage of birth weight (BW) on day of life (DOL) 3 (hours 73–96).

Secondary outcomes: Amount of enteral feeding, total fluid prescription, average urinary output, and minimal serum sodium concentration within the first 3 days of life. Comparison of central and peripheral Doppler parameters such as LVO (Left ventricular Output), RVO (Right Ventricular Output), Pulsatility Index (PI), Resistance Index (RI), V_Max_ (velocity of maximum arterial pressure) and flow of cerebral, mesenteric and renal arteries with 3 evaluations in the first 3 days of life: within the first 24 h (Time Point 1), 25–48 h (Time Point 2) and 73–96 h of life (Time Point 3).

Perinatal characteristics were collected and compared: GA, BW, gender, single or multiple pregnancy, mode of delivery, delayed cord clamping defined as >60s. As diagnosed by obstetrician: pregnancy-induced hypertension (PIH), gestational diabetes mellitus (GDM), clinical chorioamnionitis, presence of any abnormal antenatal Doppler findings such as absent or reverse end-diastolic uterine artery flow.

### Inclusion criteria

Moderately preterm to term infants (≥32 weeks of gestation) admitted to Foothills Medical Center (FMC) tertiary care NICU in Calgary, Alberta, Canada, and on any respiratory support (minimum 1 l/minute via nasal cannula, including 21%) at age 4 h of life. Parental written consents were obtained.

### Exclusion criteria

Any known congenital organ malformation or condition. Known or suspected meconium aspiration, congenital pneumonia, or pulmonary hemorrhage.

### Operational classification as RDS versus TTN

Classification was defined a priori, and the designation was consensus-based and derived from clinical, sonographic, and radiological investigations according to CNN definitions [1]:.

RDS: Babies requiring respiratory support > 24 h, intubation, surfactant administration (but not for meconium aspiration, pneumonia, or pulmonary hemorrhage), or FiO_2_ > 25% for a minimum of 24 h. CXR at day 1–2 reports RDS or hyaline membrane disease (HMD). Lung ultrasound was consistent with RDS.

TTN: Requiring any kind of respiratory support (nasal cannula with humidified low or high flow, or CPAP) for a minimum of 4 and a maximum of 24 h, and no surfactant or intubation. Lung ultrasound was consistent with TTN.

### Study procedures

Clinical information obtained from infant and maternal charts. Weight change calculated in percentage from BW at time points 2 and 3; Any **s**erum sodium levels obtained for clinical reasons during the first 3 DOL, minimum value; Total Fluid Intake (TFI) as prescribed as ml per kilogram per day; urinary output observed over at least 4, maximum 12 h, before time point 3. Heart rate, SpO2, blood pressure by non-invasive oscillatory appropriate-sized cuff measurement, and kind of respiratory support at each time point. All patients were either cared for in an incubator or open cot with body temperature monitored, ambient temperature was adjusted to maintain normothermia according to unit’s policy.

Ultrasound of the lung was completed with lung ultrasound scores calculated with a range from 0 to 18, as previously described [13]. Doppler findings of Patent Ductus Arteriosus (PDA: none, small, at least moderate in size), and calculation of left and right ventricular cardiac outputs (LVO and RVO), Aortic and Pulmonary Artery maximum velocities as previously described [14]; cerebral (anterior cerebral artery), gut (superior mesenteric artery) and renal artery perfusion parameters (RI, PI, V_Max_, and perfusion) at each time point as previously described [14]. Calculation of Cardiac Output (CO): Annular diameter (D) of aortic and pulmonic valves measured from the four-chamber view. Cross-sectional area (CSA) of the annulus derived via formula π x (D/2)^2^, then a calculation of stroke volume as SV = TVI x CSA, where TVI is time velocity integral. Indexed CO will be derived as SV x HR/ weight [15]. Pulsatility Index = PI (peak systolic - end-diastolic velocities/mean flow velocity); Resistance index = RI (peak systolic – end-diastolic velocities/peak systolic velocities) [14].

### Ethics statement

Institutional Research Ethics Board approval was obtained (REB17–2344). Written informed voluntary consent was provided by all participants’ caregivers before enrolment.

### Statistical analysis

Based on a test for difference in means between RDS and TTN groups, assuming a standard deviation of 1.25 for the variability of the weight loss, with a population sample size of 10 in each group, and at a significance level alpha of 0.05, considering the clinically important difference of 2% in weight loss between the two groups, we would have 80% power to detect such a difference. Descriptive analyses regarding the demographic characteristics of the study were performed on two groups. Mean ± standard deviation (SD) was provided for normally distributed continuous variables while the median (interquartile range, IQR, 25 to 75%) for non-normally distributed. Absolute and relative frequencies (percent) were reported for categorical variables. The distributions of continuous variables were examined for skewness and normality using Kolmogorov-Smirnov tests. Linear mixed-effects model and generalized estimating equations model were used to assess differences of vital signs, central and peripheral hemodynamic between two groups. The statistical analyses were performed by using SAS Enterprise 7.15 (SAS Institute, Cary, NC), and a two-tailed *p*-value of less than 0.05 was deemed significant.

## Results

**Fig. 1:**
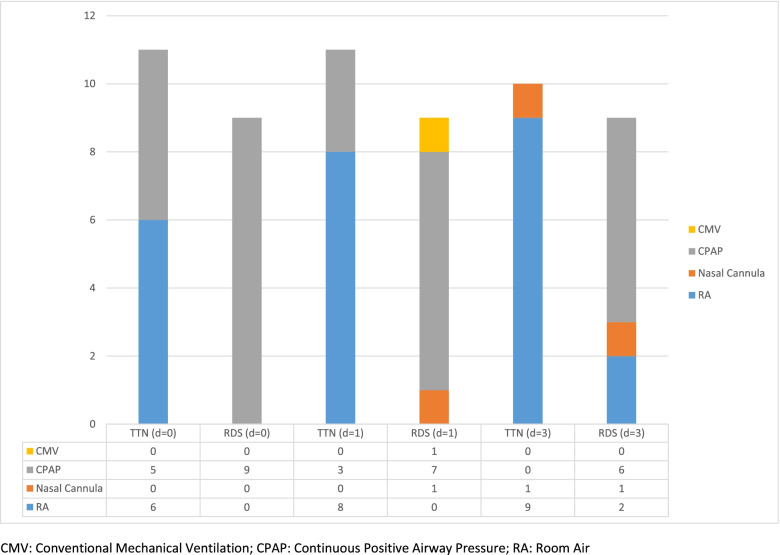
Respiratory Support during the study. CMV: Conventional Mechanical Ventilation; CPAP: Continuous Positive Airway Pressure; RA: Room Air

There were 21 babies included in this study, with 10 babies diagnosed as RDS, and 11 as TTN. GA was similar in both groups, with 33.2 +/− 1 weeks for RDS, and 34.7 +/− 3 weeks for TTN (Table 1). Mean BW was lower and less variable in the RDS group with 1768 +/− 352 g, compared to the TTN group with 2583 +/− 1013 g (*p* = 0.03, Table 1). There was no difference in gender, multiples, mode of delivery, PIH, GDM, clinical chorioamnionitis, abnormal antenatal Doppler findings, or rate of DCC between the two groups (Table 1). One baby from the TTN group had culture-positive sepsis with CONs, and no baby died. There was no difference in sepsis or empiric antibiotics between the two groups (Table 2). We found no difference in lung ultrasound scores between the 2 groups (Table 1). However, most patients in the RDS group had already received surfactant by the time of assessment and hence were excluded from lung scoring. A summary of patient respiratory support is shown in Fig. 1. Six out of the 10 (60%) patients in the RDS group, and no one in the TTN group received surfactant. Weight change from BW to time point 3 was significantly less in RDS babies with minus 2.8 +/− 2.7%, versus in TTN babies with minus 5.6+/− 3.4% (*P* = 0.003, Table 2).

**Table 1:** Demographics

	TTN (*N* = 11)	RDS (*N* = 10)	*P*-value
	N (%)	N (%)	
Male	9 (81.8)	4 (40.0)	0.06
GA (weeks) (mean ± SD)	34.7 ± 3	33.2 ± 1	0.15
BW (grams) (mean ± SD)	2583 ± 1013	1768 ± 352	0.03
Multiple Birth	3 (27.3)	5 (50.0)	0.2
Cesarean Section	7 (63.6)	9 (90.0)	0.16
PIH	3 (27.3)	2 (20.0)	0.37
GDM	2 (18.2)	1 (10.0)	0.41
Abnormal antenatal Dopplers	0 (0)	3 (30.0)	0.09
Clinical chorioamnionitis	3 (27.3)	1 (10.0)	0.25
DCC	8 (72.7)	6 (60.0)	0.3
Lung ultrasound score (mean ± SD)	2.3 ± 2.5	3.5 ± 4.1^a^	0.61

*GA* Gestational age, *BW* Birthweight, *PIH* Pregnancy-induced hypertension, *GDM* Gestational diabetes mellitus, *DCC* Delayed cord clamping; ^a^only those who did not receive surfactant

**Table 2:** Clinical characteristics and outcomes

	TTN (*N* = 11)	RDS (*N* = 10)	*P*-value
	Mean ± SD	Mean ± S D	
Sepsis; n (%)	1 (9.1)	0 (0)	0.52
Initial empiric antibiotics; n (%)	7 (63.6)	5 (50.0)	0.28
Surfactant; n (%)	0 (0)	6 (60.0)	< 0.01
Wt. change from BW at time point 1 (%)	−2.5 ± 3.9^a^	0.6 ± 3.9	0.003*
Wt. change from BW at time point 2 (%)	−5.0 ± 5.1^b^	−2.1 ± 2.2	
Wt. change from BW at time point 3 (%)	− 5.6 ± 3.4^a^	−2.8 ± 2.7	
U.O before time point 3 (ml/kg/hr)	1.4 ± 0.9	2.3 ± 1.6	0.09
Min. Na before time point 2 (mmol/L)	137.1 ± 3.4^a^	136.7 ± 4.1	0.82
TFI at time point 1 (ml/kg/d)	63.2 ± 9.0	65.0 ± 7.1	0.62
TFI at time point 2 (ml/kg/d)	75.6 ± 16.7^b^	84.0 ± 9.7	0.19
TFI at time point 3 (ml/kg/d)	120 (110, 135)^b^	120 (110, 130)	1.000**
Feeds at time point 1 (ml/kg/d)	33.6 ± 15.2	27.8 ± 19.2^b^	0.46
Feeds at time point 2 (ml/kg/d)	66.7 ± 13.5^b^	38.9 ± 22.0^b^	< 0.01
Feeds at time point 3 (ml/kg/d)	116.1 ± 17.3^b^	79.2 ± 45.0	0.03

Time point 1: first 24 h of life; Time point 2: 25-48 h of life; Time point 3: 73-96 h of life

*Wt* Weight, *BW* Birth weight, *U.O* Urine output, *Min* Minimum, *TFI* Total fluid intake

* mixed linear model; ^a^*N* = 10; ^b^*N* = 9

Babies with RDS were fed significantly less enteral feeds at both time points 2 and 3 with 38.9 and 79.2mls/kg/day respectively, compared to babies with TTN who were fed 66.7 and 116mls/kg/day, respectively (*P* < 0.01 and *P* = 0.03, Table 2). Urinary output showed a trend to be higher in RDS versus TTN (*P* = 0.09); there was no difference of TFI, nor minimal serum sodium concentration between the two groups (Table 2).

Vital signs and central Dopplers are summarized in Table 3 with expected physiological changes over time and no difference between the two groups: Blood pressure and both LVO and RVO are increasing with time. Ao_Max_ and PA_Max_ show no difference between the two groups, but there is a slightly higher number of PDA in the RDS group. None of our patients were treated for PDA. There was no difference in Doppler values for all renal parameters (Table 4). ACA V_Max_ was lower in RDS compared to TTN patients (*P* = 0.03, Table 4). There also was a trend to higher flow (*P* = 0.07), but no difference in ACA PI or RI. SMA RI was higher in RDS compared to TTN (*P* = 0.02, Table 4). There also was a trend for lower PI as well (*P* = 0.08), but no difference in SMA V_Max_ or flow between the groups.

**Table 3:** Comparison of vital signs and central hemodynamic parameters between two groups

Parameter	Day	TTN (***N*** = 11)	RDS (***N*** = 10)	***P***-value
**HR (/min)**	**0**	**144.27 ± 18.91**	**143.78 ± 12.79**	
	**1**	**144.64 ± 27.43**	**147.67 ± 19.51**	**0.63**
	**3**	**139.7 ± 28.74#**	**145.33 ± 15.12**	
	**0**	**57.73 ± 9.25**	**56.22 ± 10.17**	
**Systolic BP (mmHg)**	**1**	**58.1 ± 12.36#**	**57.22 ± 6.91**	**0.86**
	**3**	**66.8 ± 18.27#**	**67.5 ± 10.6**	
	**0**	**36.36 ± 11.76**	**32.89 ± 7.61**	
**Diastolic BP (mmHg)**	**1**	**37 ± 7.79#**	**33 ± 6.75**	**0.35**
	**3**	**43.9 ± 18.82#**	**43 ± 6.93**	
	**0**	**42.73 ± 10.28**	**41 ± 8.02**	
**BP mean (mmHg)**	**1**	**43.8 ± 9.02#**	**41.11 ± 6.74**	**0.66**
	**3**	**50.9 ± 18.01#**	**51.5 ± 7.45**	
	**0**	**96.4 ± 2.7**	**94 ± 4.1**	
**SpO2**	**1**	**98.1 ± 1.7#**	**96.6 ± 3.4**	**0.13**
	**3**	**96.2 ± 2.1#**	**96.7 ± 2.4**	
	**0**	**262.7 ± 94.7**	**237.9 ± 67.4**	
**LVO (ml/kg/min)**	**1**	**261.2 ± 105.1#**	**250.9 ± 105.3**	**0.62**
	**3**	**226 ± 101#**	**223.8 ± 87**	
	**0**	**1.43 ± 2.07#**	**0.8 ± 0.16**	
**Ao**_**Max**_ **(m/s)**	**1**	**0.76 ± 0.12**	**0.77 ± 0.13**	**0.35**
	**3**	**0.72 ± 0.14#**	**0.71 ± 0.15**	
	**0**	**370.8 ± 200.8**	**255.7 ± 150**	
**RVO (ml/kg/min)**	**1**	**406.2 ± 272.8**	**430.8 ± 164.3**	**0.46**
	**3**	**363.5 ± 200#**	**338.4 ± 127.1**	
	**0**	**0.8 ± 0.1**	**0.7 ± 0.2**	
**PA**_**Max**_ **(cm/s)**^a^	**1**	**0.8 ± 0.1**	**0.8 ± 0.2**	**0.11**
	**3**	**0.8 ± 0.2#**	**0.7 ± 0.1**	
	**0**	**3 (27.3)**	**5 (55.6)**	
**PDA present**	**1**	**0#**	**1 (11.1)**	**0.23**
	**3**	**0##**	**0**	

*HR* Heart Rate, *BP* Blood Pressure, *SpO2* Oxygen saturation, *LVO* Left ventricular output, *Ao*_*Max*_ Maximum aortic velocity, *RVO* Right ventricular output, *PA*_*Max*_ Maximum pulmonary velocity, *PDA* Patent Ductus Arteriosus

For continuous normality variables, mean ± SD, Linear Mixed Model was used

^a^For categorical variable, n (%), General Estimating Equation Model was used

Missing data: #*N* = 10; ##*N* = 7

**Table 4:** Peripheral hemodynamic variables between two groups

Parameter	Day	TTN (*N* = 11)	RDS (*N* = 10)	*P*-value
ACA PI	0	1.38 ± 0.66	1.46 ± 0.51	
	1	1.16 ± 0.28^a^	1.16 ± 0.3	0.66
	3	1.27 ± 0.26 ^a^	1.25 ± 0.2^b^	
	0	0.71 ± 0.19	0.81 ± 0.15	
ACA RI	1	0.65 ± 0.08^a^	0.67 ± 0.11	0.21
	3	0.69 ± 0.08^a^	0.69 ± 0.06^b^	
	0	31.11 ± 10.9	22.23 ± 11.98	
ACA_Vmax_ (cm/s)	1	27.96 ± 8.51^a^	26.61 ± 8.86	**0.03**
	3	37.47 ± 12.6^a^	29.46 ± 7.29^b^	
	0	9.87 ± 6.25	5.51 ± 4.15	
ACA Flow (ml/kg/min)	1	13.54 ± 9.11^a^	9.11 ± 7.14	0.07
	3	12.34 ± 7.09^a^	13.66 ± 10.29^b^	
	0	1.36 ± 0.28	1.72 ± 0.87	
SMA PI	1	1.32 ± 0.42	1.51 ± 0.4	0.08
	3	1.31 ± 0.42^a^	1.44 ± 0.33	
	0	0.69 ± 0.07	0.76 ± 0.17	
SMA RI	1	0.68 ± 0.1	0.78 ± 0.11	**0.02**
	3	0.67 ± 0.11^a^	0.7 ± 0.1	
	0	61.1 ± 19.78	64.95 ± 32.02	
SMA_Vmax_ (cm/s)	1	60.79 ± 16.01	52.69 ± 21.62	0.31
	3	51.4 ± 12.05^a^	48.43 ± 10.63	
	0	40.65 ± 37.2	27.6 ± 19.31	
SMA Flow (ml/kg/min)	1	34.66 ± 39.32	23.54 ± 11.24	0.28
	3	20.81 ± 10.72^a^	25.49 ± 8.3	
	0	1.87 ± 0.78	4.97 ± 7.98	
Renal PI	1	1.56 ± 0.5	2.29 ± 2.06	0.99
	3	1.53 ± 0.73^a^	1.65 ± 0.45	
	0	0.78 ± 0.14	0.87 ± 0.1	
Renal RI	1	0.78 ± 0.15	0.79 ± 0.17	0.24
	3	0.74 ± 0.14^a^	0.77 ± 0.1	
	0	26.67 ± 11.93	29.97 ± 12.53	
Renal_Vmax_ (cm/s)	1	30.41 ± 8.23	40.09 ± 10.63	0.12
	3	36.71 ± 10.62^a^	36.94 ± 8.76	
	0	7.23 ± 4.62	5.9 ± 4.77	
Renal Flow (ml/kg/min)	1	10.66 ± 8.1	17.42 ± 9.72	0.17
	3	11.95 ± 11.41^a^	16.78 ± 14.48	

*PI* Pulsatility Index, *RI* Resistance Index, *ACA* Anterior Cerebral Artery, *Vmax* Maximum Velocity, *SMA* Superior Mesenteric Artery

For continuous normality variables, mean ± SD, Linear Mixed Model was used

Missing data: ^a^*N* = 10; ^b^*N* = 8

## Discussion

In our comparative cohort study of a group of neonates with RDS versus TTN, infants in the RDS group had less weight loss on all three time points than the TTN group, despite having been prescribed similar TFI (Table 2). This occurred despite those with RDS being significantly lower in BW than their TTN counterparts which may put them at higher risk of weight loss [16]. This concurs with previous clinical observations and the recommended approach to RDS treatment with fluid restriction to counteract the disease [17]. Furthermore, enteral feedings were significantly less advanced on time points 2 and 3 for the infants with RDS, compared to those with TTN (Table 2). The RDS cohort was significantly smaller in BW and had a PDA seen on ultrasound, albeit none treated, in 55.6% compared to 27.3% in TTN (*p* = 0.23, Table 3). These differences may likely explain the higher degree of respiratory support the RDS group required, cautioning the clinician to advance feeds slower (Fig. 1) [18]. Based on these differences and the fact that 60% in our RDS cohort received surfactant, we ascertained a reasonable group distinction between the two entities. However, some overlap between the 2 entities cannot be excluded. Interestingly, our cohorts were not different in GA, despite RDS being generally considered a disease predominantly of the preterm neonate, and TTN in the near-term and term population. Unfortunately, due to the timing of the lung ultrasounds (after surfactant administration in all of those with surfactant), we were not able to differentiate the two conditions based on the lung ultrasound score results (Table 1).

We could not show a different urinary output or different minimal serum sodium concentration between the two groups. This may be due to the small random time frame we used to calculate urinary output. Furthermore, weight loss is a more inclusive marker of fluid loss than just urinary output as it includes insensible losses. Serum sodium concentration is dependent on care providers’ supplementation, usually with intravenous fluid in the first days of life, and any potential dilution hyponatremia would have been counteracted by parenteral nutrition electrolyte prescription. All values reported in our cohort were within normal limits for newborn infants, with 136.7 ± 4.1 mmol/l for RDS and 137.1 ± 3.4 mmol/l for TTN [19].

We found no difference in vital signs nor in central Doppler parameters between the RDS and TTN groups (Table 3). Expected vital sign changes around the time of adaptation were occurring as expected with increasing heart rates and blood pressures, as well as LVO and RVO [20]. The higher RVO, compared to LVO, is likely explained by some shunting across a Persistent Foramen Ovale and PDA, which almost certainly persist during this early time of neonatal transition. There were more PDA seen in our RDS cohort, compared to the TTN, which is expected as RDS is a known risk factor for PDA [21]. In keeping with our more mature cohort of moderately preterm to term infants, none of our babies required therapeutic PDA closure. Furthermore, there was no difference in any of the renal Doppler parameters interrogated during neonatal transitioning in the first 3 days of life (Table 4). However, other organ Doppler variables showed some interesting yet unexpected findings in our exploratory analysis: The ACA V_Max_ was significantly lower in the RDS compared to the TTN group (Table 4). However, no difference was seen in ACA PI, RI, or flow (Table 4). With more pulmonary disease ongoing, there is expected to be blood shunted away from the peripheral organs, especially through a very proximal open PDA which may explain this. Furthermore, the SMA showed a higher resistance pattern with higher RI in RDS compared to TTN, with no difference in PI, V_Max_, or intestinal blood flow. We speculate that the lower SMA resistance to flow in those with TTN may be in response to the higher enteral feeding volume in this group, compared to RDS (Table 2). It is known that SMA blood flow patterns may be influenced by feedings [22]. Our peripheral Doppler values are comparable to previous findings from our group [14]: Compared to healthy transitioning babies without respiratory issues measured within the first 24 h of life, patterns of both ACA, renal, and SMA PI, RI continue to trend down in our current slightly older population, whereas ACA flow increases. Interestingly, renal flow seems to continue the trend of increasing over time from 3.8–4.6 ml/kg/min within the first day to 5.9–17.4 ml/kg/min over days 2 and 3 in the current cohort [14]. Overall, our findings may be exploratory and in a small cohort but opens the floor to examine perinatal flow patterns in larger neonatal cohorts.

### Study limitations

Small sample size. There may have been some overlap between RDS and TTN in those who did not receive surfactant. Limitations of Doppler assessment apply. Timing of ultrasound to feeds could not be made standard for practical reasons.

## Conclusion

In our cohort of ≥32 weeks moderately preterm to term neonates during the first 3 days of life, those with RDS had less weight loss and were prescribed less enteral feeds than those with TTN despite similar total fluid intake. There was no difference in renal or central Doppler parameters between the RDS and TTN groups. Cerebral artery V_Max_ was lower in RDS and mesenteric artery resistance index was higher, compared to TTN, suggesting different blood flow patterns according to disease and may imply grounds for differential management.

## Data Availability

Raw data available upon reasonable request from the corresponding author, Amelie.Stritzke@albertahealthservices.ca

## Abbreviations

ACA: Anterior cerebral artery
Ao_Max_: Maximum aortic velocity
BW: Birth Weight
CNN: Canadian Neonatal Network
CO: Cardiac Output
CONs: Coagulase-negative Staphylococcus
CPAP: Continuous Positive Airway Pressure
CSA: Cross-sectional Area
CXR: Chest X ray
D: Diameter
DCC: Delayed Cord Clamping
DOL: Day of Life
FMC: Foothills Medical Center
GA: Gestational Age
GDM: Gestational diabetes mellitus
HMD: Hyaline Membrane Disease
HR: Heart Rate
IQR: Interquartile range
LVO: Left Ventricular Output
NICU: Neonatal Intensive Care Unit
PAH: Para-aminohippurate
PA_Max_: Maximum Pulmonary Artery Velocity
PDA: Patent Ductus Arteriosus
PI: Pulsatility Index
PIH: Pregnancy-induced Hypertension
RDS: Respiratory Distress Syndrome
REB: Research Ethics Board
RI: Resistance Index
RVO: Right Ventricular Output
SD: Standard Deviation
SMA: Superior mesenteric artery
SpO_2_: Oxygen saturation
SV: Stroke Volume
TFI: Total Fluid Intake
TTN: Transient Tachypnea of the Newborn
TVI: Time Velocity Integral
V_Max_: Maximum velocity

## References

[1] Canadian neonatal network (CNN). Annu Rep http://www.canadianneonatalnetworkorg/Portal/LinkClickaspx?fileticket=PJSDwNECsMI%3d&tabid=39. 2016;Accessed 1 May 2021.

[2] Mally P, Hendrick-Munoz K, Bailey S (2013). Incidence and etiology of late preterm admissions to the neonatal intensive care unit and its associated respiratory morbidities when compared to term infants. Am J Perinatol.

[3] Guglani L, Lakshminrusimha S, Ryan R (2008). Transient tachypnea of the newborn. Pediatr Rev.

[4] Brusa G, Savoia M, Vergine M, Bon A, Copetti R (2015). Neonatal lung Sonography: Interobserver agreement between physician interpreters with varying levels of experience. J Ultrasound Med.

[5] Sweet D, Carnielli V, Greisen G, Hallman M, Ozek E (2017). European consensus guidelines on the Management of Respiratory Distress Syndrome - 2019 update. Neonatology..

[6] Stevens T, Finer N, Carlo W, Szilagyi P, Phelps D (2014). Respiratory outcomes of the surfactant positive pressure and oximetry randomized trial (SUPPORT). J Pediatr.

[7] Stritzke A, Thomas S, Amin H, Fusch C, Lodha A (2017). Renal consequences of preterm birth. Mol Cell Pediatr.

[8] Heaf D, Belik J, Spitzer A, Gewitz M, Fox W (1982). Changes in pulmonary function during the diuretic phase of respiratory distress syndrome. J Pediatr.

[9] Torrado A, Guignard J, Prod'hom L, Gautier E (1974). Hypoxaemia and renal function in newborns with respiratory distress syndrome (RDS). Helv Paediatr Acta.

[10] Guignard J, Torrado A, Mazouni S, Gautier E (1976). Renal function in respiratory distress syndrome. J Pediatr.

[11] Langman CB, Engle WD, Baumgart S, Fox WW, Polin RA (1981). The diuretic phase of respiratory distress syndrome and its relationship to oxygenation. J Pediatr.

[12] Hooper S, Kitchen M, Polglase G, Roehr C, Te Pas A (2018). The physiology of neonatal resuscitation. Curr Opin Pediatr.

[13] De Martino L, Yousef N, Ben-Ammar R, Raimondi F, Shankar-Aguilera S, De Luca D. Lung ultrasound score predicts surfactant need in extremely preterm neonates. Pediatrics. 2018;142(3). 10.1542/peds.2018-0463 30108142

[14] Stritzke A, Murthy P, Kaur S, Kuret V, Liang Z, Howell S, Tyberg J. Arterial flow patterns in healthy transitioning near-term neonates. BMJ Paediatr Open. 2019;3(1). 10.1136/bmjpo-2018-000333 PMC6422249 30957024

[15] Lewis JFKL, Nelson JG, Limacher MC, Quinones MA (1984). Pulsed Doppler echocardiographic determination of stroke volume and cardiac output: clinical validation of two new methods using the apical window. Circulation..

[16] Christensen R, Henry E, Kien T, Stree J (2005). Pattern of daily weights among low birth weight neonates in the neonatal intensive care unit: data from a multi-hospital health-care system. J Perinatol.

[17] Diaz F, Nunez M, Pino P, Erranz B, Cruces P (2018). Implementation of preemptive fluid strategy as a bundle to prevent fluid overload in children with acute respiratory distress syndrome and sepsis. BMC Pediatr.

[18] Oezkan H, Erdeve O, Kutman GK, H. (2018). Turkish neonatal society guideline on the management of respiratory distress syndrome and surfactant treatment. Turk Pediatr Ars.

[19] Manual Merck. https://www.merckmanuals.com/en-ca/professional/resources/normal-laboratory-values/blood-tests-normal-values. Accessed 14 Apr 2021.

[20] Noori S, Wlodaver A, Gottipati V, McCoy M, Schultz D (2012). Transitional changes in cardiac and cerebral hemodynamics in term neonates at birth. J Pediatr.

[21] Sankar M, Bhombal S, Benitz W (2019). PDA: to treat or not to treat. Congenit Heart Dis.

[22] Maruyama K, Fujiu T, Inoue T, Koizumi A, Inoue F (2013). Feeding interval and postprandial intestinal blood flow in premature infants. Pediatr Int.

