# Correction to: Fluid handling and blood flow patterns in neonatal respiratory distress syndrome versus transient tachypnea: a pilot study

**DOI:** 10.1186/s12887-022-03110-x

**Published:** 2022-01-19

**Authors:** Rana Ismail, Prashanth Murthy, Ayman Abou Mehrem, Zhiying Liang, Amelie Stritzke

**Affiliations:** 1 Section of Neonatology, Department of Pediatrics, Alberta Health Services, University of Calgary, Cumming School of Medicine, Calgary, Canada; 2 University of Calgary, Cumming School of Medicine, Libin Cardiovascular Institute of Alberta, Calgary, Canada

**Correction to: BMC Pediatr 21, 541 (2021)**

**https://doi.org/10.1186/s12887-021-03025-z**

Following the publication of the original article [1], it has been noticed that the author names have been captured incorrectly. The given and family names were interchanged. The correct names are provided in the authorgroup section above and the reference entry below.

The original article has been corrected.
